# Settleable Dust and Bioburden in Portuguese Dwellings

**DOI:** 10.3390/microorganisms8111799

**Published:** 2020-11-16

**Authors:** Carla Viegas, Marta Dias, Beatriz Almeida, Estela Vicente, Liliana Aranha Caetano, Elisabete Carolino, Célia Alves

**Affiliations:** 1 H&TRC-Health & Technology Research Center, ESTeSL-Escola Superior de Tecnologia da Saúde, Instituto Politécnico de Lisboa, 1990-096 Lisbon, Portugal; martasfd@gmail.com (M.D.); beatrizltalmeida1@gmail.com (B.A.); liliana.caetano@estesl.ipl.pt (L.A.C.); etcarolino@estesl.ipl.pt (E.C.); 2 NOVA National School of Public Health, Public Health Research Centre, Universidade NOVA de Lisboa, 1600-560 Lisboa, Portugal; 3 Comprehensive Health Research Center (CHRC), 1169-056 Lisbon, Portugal; 4 Department of Environment and Planning, Centre for Environmental and Marine Studies (CESAM), University of Aveiro, 3810-193 Aveiro, Portugal; estelaavicente@ua.pt (E.V.); celia.alves@ua.pt (C.A.); 5 Research Institute for Medicines (iMed.ULisboa), Faculty of Pharmacy, University of Lisbon, 1649-003 Lisbon, Portugal

**Keywords:** indoor air quality, dwellings, passive sampling methods, settleable dust, bioburden, azole-resistance screening, *Aspergillus* sp.

## Abstract

Monitoring campaigns in several buildings have shown that occupants exposed to contaminated indoor air generally exhibit diverse health symptoms. This study intends to assess settleable dust loading rates and bioburden in Portuguese dwellings by passive sampling onto quartz fiber filters and electrostatic dust cloths (EDCs), respectively. Settled dust collected by EDCs was analyzed by culture-based methods (including azole-resistance screening) and qPCR, targeting four different toxigenic *Aspergillus* sections (*Flavi*, *Fumigati*, *Circumdati,* and *Nidulantes*). Dust loading rates and bioburden showed higher variability in the summer season. In both seasons, *Penicillium* sp. was the one with the highest prevalence (59.1% winter; 58.1% summer), followed by *Aspergillus* sp. in winter (13.0%). Fungal contamination increased in the winter period, while bacterial counts decreased. *Aspergillus* sections *Circumdati* and *Nidulantes*, detected in voriconazole supplemented media, and *Aspergillus* sections *Fumigati* and *Nidulantes*, detected by molecular tools, were found in the winter samples. This study reinforces the importance of applying: (a) Passive sampling methods in campaigns in dwellings; (b) two different culture media (MEA and DG18) to assess fungi; (c) in parallel, molecular tools targeting the most suitable indicators of fungal contamination; and (d) azole resistance screening to unveil azole resistance detection in fungal species.

## 1. Introduction

According to the World Health Organization (WHO), 4.3 million people die each year from exposure to domestic air pollution. Presently, people spend more than 90% of the day indoors in their own dwellings or in workplace [1,2], so it is of utmost importance to study indoor air quality (IAQ).

Organic dust consists mainly of particulate matter with microbial, vegetable, or animal origin. Its specific agents include viruses, bacteria, gram negative endotoxins, actinomycetes, fungi, mycotoxins, algae or plant cells, enzymes and proteins from plants or animals, antibiotics or other products from other processes, insects, and mites (and their fragments and particles) [3,4]. Among organic dust, bioaerosols are usually defined as particulate matter with biological origin, such as pollen, plant fibers, and microorganisms. Exposure to bioaerosols can lead to a wide range of adverse health effects [5,6,7,8]. Fungi and bacteria present in bioaerosols are often called as the bioburden and should be well characterized [9].

IAQ studies in several buildings have shown that occupants exposed to contaminated air generally exhibit signs of lethargy or fatigue, headaches, dizziness, vomiting, difficulty in concentrating, and other symptoms [10]. Among the monitoring of other environmental parameters, the collection of particulate matter (PM) inside buildings is commonly used for studies linking human health to disease [11]. Additionally, it is apparent that not only quantitative but also qualitative aspects (species present) of the microbial exposure may be important to understand agents and the mechanisms causing health outcomes in building occupants [12].

Previous studies have pointed out *Cladosporium, Penicillium,* and *Aspergillus* as the most prevalent genera indoors [13,14]. *Micrococcus* sp., *Staphylococcus auricularis*, and the gram-negative bacteria *Bacillus* sp. have been documented as dominant among bacteriota [2,13].

Several studies reporting a wide range of environmental factors that influence bioburden indoors [6,15,16,17,18] have indicated that microbial sampling should be achieved by passive methods, as a complement or alternative to the more conventional air sampling techniques [19,20,21]. Indeed, passive methods allow reporting the contamination of an extended period of time (ranging from days to several months), while air samples can only reproduce the load for a shorter period of time (mostly minutes) [22].

Passive monitoring of settleable dust onto filters has been used both indoors and outdoors [23,24,25]. It is a cost-effective and simpler alternative to active sampling, allowing the simultaneous obtaining of a larger number of samples in various locations. On the other hand, passive sampling is less disturbing for indoor occupants since it does not rely on noisy pumps.

Electrostatic dust cloths (EDC) are an inexpensive passive sampling method comprising an electrostatic polypropylene cloth inside an open sterilized petri dish [22,26,27]. The cloth consists of electrical fibers that increase the retention of particles [22,27] and, if located on an elevated surface, it allows the efficient collection of the dust present in the air [27,28].

In previous studies carried out in Portugal, passive sampling has, moreover, allowed the recovery of fungal contaminants with reduced susceptibility to azoles in distinct indoor environments [29,30,31]. The emergence in the environment of human pathogenic fungal species, such as *Candida* sp. and *Aspergillus fumigatus*, with reduced susceptibility to the antifungal drugs, raises concern regarding the limited therapeutic arsenal available to treat fungal infections that might become severe, even mortal, particularly in individuals with some type of immune impairment [32,33,34]. This phenomenon calls for worldwide surveillance of fungal resistance both indoors and outdoors [35,36].

In this study, the seasonal deposition rates of total settleable dust and bioburden in dwellings was assessed by passive sampling. The suitability of EDC as screening method to characterize bioburden was also explored. Additionally, the study comprised the molecular detection of toxigenic fungal species and the analysis of antifungal resistance profiles.

## 2. Materials and Methods

### 2.1. Location of the Studied Dwellings

**Figure 1:**
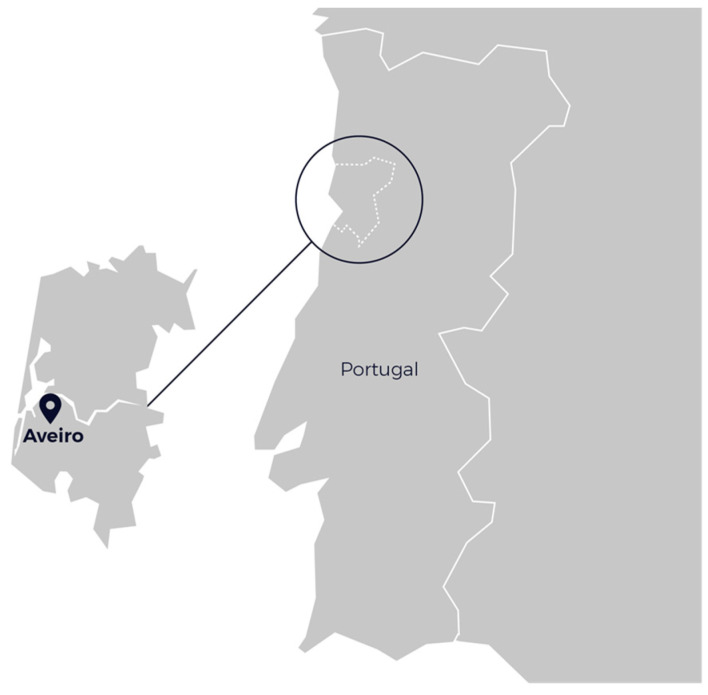
Location of the district of Aveiro.

This study was conducted in 23 naturally ventilated dwellings located in the district of Aveiro, Portugal (Figure 1). Simultaneous samplings were made in 3 rooms of each house: Kitchen, bedroom, and living room. Table 1 summarizes the main characteristics of each dwelling.

### 2.2. Settled Dust Sampling

Two 47 mm diameter quartz fiber filters (Pallflex^®^ Putnam, CT, USA) were exposed in uncovered petri dishes (Analyslide^®^ Pall, München, Germany), which were placed side by side to collect settleable particulate matter in the rooms of each home in two different seasons. The filters were placed at a height of approximately 1.5 m above ground level and exposed to dust fall for about 1 month. Sampling took place approximately between 20 May and 20 June 2017 (summer campaign) and between 20 January and 27 February 2018 (winter campaign). The gravimetric quantification was performed with a microbalance (RADWAG 5/2Y, Radom, Poland) after conditioning the filters for 24 h in a room with constant humidity (50%) and temperature (20 °C) in accordance with the European Standard EN14907:2005. Filter weights were obtained from the average of six consecutive measurements with variations less than 0.02%.

To assess bacterial and fungal contamination indoors, dust was also collected through a passive method using an Electrostatic Dust Collector (EDC), which comprises an electrostatic polypropylene cloth [26]. A total of 79 EDC was collected in summer and 78 in winter. EDCs were placed in large petri dishes (surface expose area of 154 cm^2^) in parallel with the two small petri dishes with quartz filters used for gravimetric quantification. The 3 devices were exposed to dust fall for the same time. After transport in refrigerated conditions (<4 °C), EDCs were then used for the bioburden assessment.

### 2.3. Electrostatic Dust Cloth Extraction and Bioburden Characterization

Settled dust collected by EDCs was analyzed by culture-based methods and qPCR, targeting 4 different toxigenic *Aspergillus* sections (*Flavi*, *Fumigati*, *Circumdati*, and *Nidulantes*). The target fungi were selected upon the classification as indicators of harmful fungal contamination through culture-based methods [37].

EDC samples were subjected to extraction and bioburden characterized by culture-based methods, as previously described [22,38]. EDCs were washed and plated onto 2% malt extract agar (MEA) (Frilabo, Maia, Portugal) with 0.05 g/L chloramphenicol media, dichloran glycerol (DG18) (Frilabo, Maia, Portugal) agar-based media, tryptic soy agar (TSA) (Frilabo, Maia, Portugal) with 0.2% nystatin, and violet red bile agar (VRBA) (Frilabo, Maia, Portugal). Incubation of MEA and DG18 plates at 27 °C for 5 to 7 days and TSA and VRBA plates at 30 °C and 35 °C for 7 days, respectively, was performed. From the EDC suspension, 150 µL were additionally plated on Sabouraud dextrose agar (SDA) (Frilabo, Maia, Portugal), as well as on SDA plates supplemented with 4 mg/L itraconazole (ITR) (Frilabo, Maia, Portugal), 1 mg/L voriconazole (VOR) (Frilabo, Maia, Portugal), 0.5 mg/L posaconazole (POS) (Frilabo, Maia, Portugal), and incubated at 27 °C (adapted from the EUCAST 2020 guidelines).

Molecular identification of the different fungal species/strains was achieved by Real Time PCR (qPCR) using the CFX-Connect PCR System (Bio-Rad, Hercules, CA, USA) on each EDC. Reactions included 1× iQ Supermix (Bio-Rad, Hercules, CA, USA), 0.5 μM of each primer (Table 2), and 0.375 μM of TaqMan probe in a total volume of 20 μL. Amplification followed a three-step PCR: 50 cycles with denaturation at 95 °C for 30 s, annealing at 52 °C for 30 s, and extension at 72 °C for 30 s. A non-template control was used in every PCR reaction. For each gene that was amplified, a non-template control and a positive control were used, consisting of DNA obtained from a reference that belonged to the culture collection of the Reference Unit for Parasitic and Fungal Infections, Department of Infectious Diseases of the Ricardo Jorge National Institute of Health. These strains have been sequenced for ITS B-tubulin and calmodulin.

### 2.4. Statistical Analysis

Data were analyzed using the statistical software SPSS V26.0 for Windows. Results were considered significant at the 5% significance level. For the characterization of the sample, frequency analysis (n, %) was used for qualitative data and mean and standard deviation for quantitative data. To test the normality of the data, the Shapiro–Wilk test was used. In order to study the relationship between bacterial and fungal counts, azole resistance, dust load, and Cq, the Spearman’s correlation coefficient was used, since the assumption of normality was not verified. The Kruskal–Wallis test was used to compare house divisions, since the assumption of normality was not confirmed. To compare the bacterial and fungal counts, azole resistance, and dust load between summer and winter, the Wilcoxon test was used, as the assumption of normality was also not observed.

## 3. Results

### 3.1. Dust Loading Rates

The highest values were registered in the three rooms of an apartment with a baby, still in the phase of changing diapers with the use talcum powder, in the kitchen of a dwelling where four cats remained full time, and in a terraced house in the vicinity of construction works (Table 3). Between summer and winter, statistically significant differences were detected in relation to dust load (z = −3.187, *p* = 0.001), with lower values in the cold season (Table 4).

The comparison between house divisions in both seasons also revealed lower dust loads in the winter period for bedrooms (z = −2.538, *p* = 0.011) and living rooms (z = −2.053, *p* = 0.040). However, in the kitchens, no statistically significant differences were detected between summer and winter (z = −1.282, *p* = 0.200).

### 3.2. Bacterial Contamination

In EDC collected in summer, the total bacteria sedimentation rates ranged from 0 to 1.42 × 10^5^ CFU/m^2^/day, while the Gram-negative bacteria varied from 0 to 3.65 × 10^3^ CFU/m^2^/day. In winter samples, total bacteria and Gram-negative bacteria were in the range 0–1.07 × 10^3^ CFU/m^2^/day and 0–8.67 × 10^2^ CFU/m^2^/day, respectively (Table 5).

### 3.3. Fungal Contamination

Fungal counts ranged from 0 to 3.18 × 10^2^ CFU/m^2^/day on MEA and from 0 to 3.72 × 10^2^ CFU/m^2^/day on DG18. *Penicillium* sp. presented the highest prevalence (1.45 × 10^3^ CFU/m^2^/day; 58.1%) on MEA media, followed by *C. sitophila* (2.48 × 10^2^ CFU/m^2^/day; 9.92%). On DG18, the highest prevalence was found for *Cladosporium* sp. (1.45 × 10^3^ CFU/m^2^/day; 46.3%), followed by *Penicillium* sp. (1.09 × 10^3^ CFU/m^2^/day; 34.9%).

In winter samples, fungal counts ranged from 0 to 2.18 × 10^2^ CFU/m^2^/day on MEA and from 0 to 3.34 × 10^2^ CFU/m^2^/day on DG18. *Penicillium* sp. presented the highest prevalence on both media (1.47 × 10^3^ CFU/m^2^/day, 59.1% MEA; 1.69 × 10^3^ CFU/m^2^/day, 52.2% DG18), followed by *Aspergillus* sp. (3.22 × 10^2^ CFU/m^2^/day, 13.0% MEA) and *Cladosporium* sp. (7.11 × 10^2^ CFU/m^2^/day; 21.9% DG18) (Table 6).

**Figure 2:**
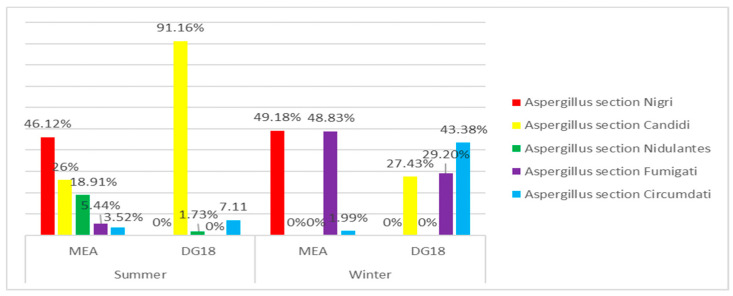
*Aspergillus* sections identified in winter and summer on EDC samples.

Among *Aspergillus* genus, section *Nigri* was found as the most prevalent in both seasons on MEA media (46.1% summer; 49.2% winter), followed by section *Candidi* in summer (26.0%) and section *Fumigati* in winter (48.8%). Regarding DG18 media, section *Candidi* presented the highest prevalence in summer (91.2%), followed by section *Circumdati* (7.11%). In winter, section *Circumdati* was identified as the most abundant (43.4%), succeeded by section *Fumigati* (29.2%) (Figure 2).

### 3.4. Azole-Resistance Screening

Azole resistance frequencies were as follows: From 43.5% (winter) to 60.9% (summer) in ITR, from 91.3% (winter) to 95.7% (summer) in VOR, and from 39.1% (summer) to 52.2% (winter) in POS. Pan-azole resistance (in homes where fungal growth was observed in the three azoles at tested concentrations) was found to be in the range from 21.7% (summer) to 30.4% (winter). Table 7 summarizes fungal burden found in each home location. Kitchens revealed the highest burdens among all tested azoles, with one exception. In fact, in wintertime, the fungal burden was higher in samples from living rooms cultivated in ITR media.

Results of identified fungal genera are presented in Table 8, organized by season, for SDA media only (which served as growth control without antimycotic), 4 mg/L itraconazole (ITR), 1 mg/L voriconazole (VOR), and 0.5 mg/L posaconazole (POS).

**Figure 3:**
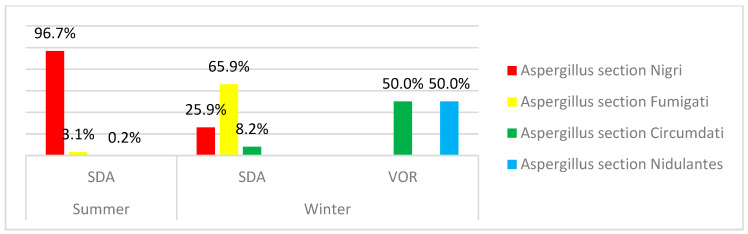
*Aspergillus* sections identified in summer and winter EDC samples by the azole screening method.

Among *Aspergillus* genera, sections *Nigri* (96.7% summer, 26.0% winter) and *Fumigati* (65.9% winter, 3.1% summer) presented the highest frequencies on SDA, whereas *Aspergillus* sections *Circumdati* and *Nidulantes* were detected in voriconazole supplemented SDA media in samples from the winter campaign (Figure 3).

### 3.5. Molecular Assessment

*Aspergillus* sections were detected by molecular tools in nine samples (9 out of 154 samples, i.e., 5.8%) in the winter season. In these nine EDCs, only one *Aspergillus* section was detected in each sample. Sections *Fumigati* and *Nidulantes* were detected in seven (4.6%, 7 out of 154 samples) and two samples (1.3%, 2 out of 154 samples), respectively (Table 9).

### 3.6. Correlation Analyses

In summer, only a significant correlation was detected between fungal counts on MEA and on DG18 (r_S_ = 0.430, *p* = 0.000), indicating that higher fungal counts on MEA is related to higher fungal counts on DG18 (Table 6).

In winter, more significant positive correlations were detected: (i) Dust loadings with bacteria counts on TSA (r_S_ = 0.397, *p* = 0.001) and fungi in azole-screening on POS (r_S_ = 0.244, *p* = 0.050); (ii) bacterial counts on TSA with bacterial counts on VRBA (r_S_ = 0.305, *p* = 0.009); (iii) fungal counts on MEA and on DG18 (r_S_ = 0.710, *p* = 0.000), and, at a lower extent, with fungal counts on ITR (r_S_ = 0.380, *p* = 0.001), VOR (r_S_ = 0.382, *p* = 0.001), and POS (r_S_ = 0.281, *p* = 0.016); (iv) fungal counts on DG18 with fungal counts on ITR (r_S_ = 0.246, *p* = 0.035) and VOR (r_S_ = 0.419, *p* = 0.000); (v) fungal counts on ITR and POS (r_S_ = 0.312, *p* = 0.006); (vi) fungal counts on VOR and on POS (r_S_ = 0.463, *p* = 0.000); and (vii) fungal counts on POS with Cq (r_S_ = 0.772, *p* = 0.015) (Table 10).

### 3.7. Comparison Analysis

**Figure 4:**
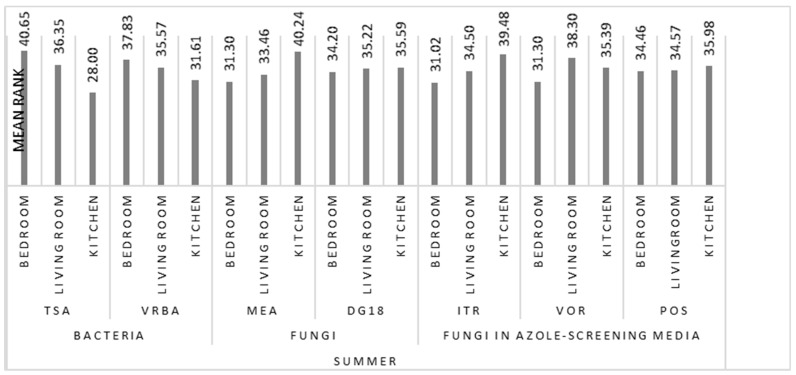
Results of the Kruskal–Wallis multiple comparisons of bacterial and fungal counts, azole, and Cq in the three sampling locations (bedroom, living room, and kitchen), in summer.

**Figure 5:**
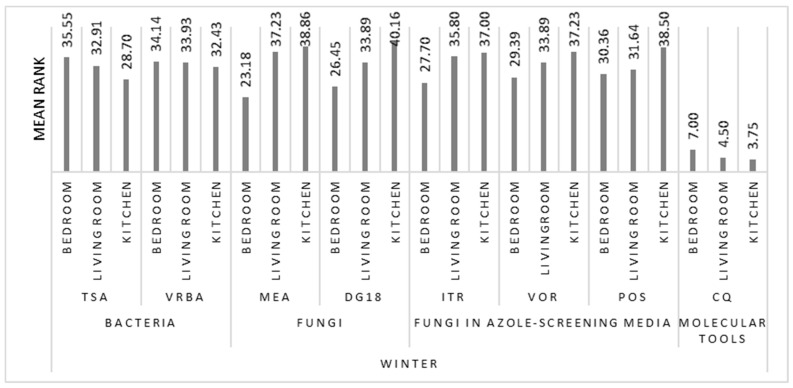
Results of the Kruskal-Wallis multiple comparisons of bacterial and fungal counts, azole, and Cq in the three sampling locations (bedroom, living room, and kitchen), in winter.

The comparison between the three sampling locations, bedroom, living room, and kitchen, both in summer and winter, revealed statistically significant differences only for fungal counts on MEA for the cold period $\left(\chi_{K-W}^{2}\left(2\right)=9.140,\;p=0.010\right)$. The application of the Kruskal–Wallis test showed significant differences between the bedroom and the other divisions of the house. Fungal counts were found to be substantially higher in kitchens and living rooms (Figure 4 and Figure 5). Furthermore, from the analysis of Figure 5, it can be observed that the trend is identical in summer and in winter, both in relation to bacterial and fungal counts and to fungal growth in azole-supplemented media.

Among the three types of geographical location of the houses (urban, rural, or suburban), no statistically significant differences were detected, either in summer or in winter, in relation to dust loadings, bacterial counts (TSA and VRBA), fungal counts (MEA and DG18), and azole resistance screening (ITR, VOR, and POS) (*p*’s > 0.05).

The comparison between seasons displayed statistically significant differences with higher values in winter for: (i) Bacterial counts on TSA (z = −6.624, *p* = 0.000), (ii) bacterial counts on VRBA (z = −2.761, *p* = 0.005), (iii) fungal counts on MEA, and (iv) fungal counts on DG18 (Table 11).

As observed for the dwellings, lower bacteria counts and higher fungal levels were detected in winter in bedrooms and living rooms. For kitchens, in the cold season, lower bacterial counts on TSA (z = −3.724, *p* = 0.000), and higher fungal counts on MEA (z = −3.389, *p* = 0.001) and DG18 (z = −3.620, *p* = 0.000) were found.

With regard to the characteristics of the dwellings (Table 1), comparisons were not possible due to the small number of observations.

## 4. Discussion

The use of the passive sampling methods in this study allowed the simultaneous collection of settleable dust, for extended periods, in several homes with wide spatial coverage and without disturbing daily life [43]. A single EDC analysis is equivalent to the sum of several air-impaction measurements, with much shorter sample collection duration, permitting a more consistent estimation of exposure [44]. Although settleable dust analysis is only a surrogate measure for airborne exposure, and differences between settled and airborne bioburden should be considered [28], with EDCs it was possible to obtain a greater fungal diversity. This situation was corroborated with *Aspergillus* sections counts, when compared to air samples collected by impaction or even with other passive methods, such as surface swabs, as it was the case in other studies [22]. Five different *Aspergillus* sections were observed in this study.

Sampling in parallel, and in duplicate, of settleable dust, whose sedimentation rates were gravimetrically determined according to an international standard, allowed a more accurate estimation of exposure levels inside the dwellings [43] and, together with the dwellings’ characteristics, can give indications about possible risks and assist in taking remedial measures.

Differences between sampling locations in the dwellings can be due to several reasons. In fact, particle deposition depends on the size of the particles, their sedimentation processes (gravity in the case of larger particles or diffusion in the case of smaller particles) [45], the amount of furniture indoors [46], the type of ventilation, and air turbulence caused by human activities [47].

The dust loading rates of the present study are lower than the values described for dwellings in arid regions, but close to those addressed in other European countries. Khoder and colleagues evaluated the loading rates of surface dust in domestic houses in an urban area of Giza, Egypt, reporting a mean value of 226 mg/m^2^/day [48]. Shraim and colleagues collected dust samples from 38 naturally ventilated houses of arid and dry climatic regions, documenting loading rates from 2.5 to 19.4 mg/m^2^/day, with a median of 8.5 mg/m^2^/week [49]. Seifert and colleagues registered mean values of 9.52 and 10.9 mg/m^2^/day in homes of the German adult and children population, respectively [50].

Overall, as in dust loading rates, bioburden presented a wider range in the summer season. This can be due to the fact that particles can act as carriers of bioburden inside dwellings through open windows [51]. Since microbial exposures may have different sources, both indoors and outdoors, the air exchange rates (AER) may influence the indoor bioaerosol levels. It has been reported that the higher the AER, the more bioaerosols enter the home, especially when the outdoor temperature is favourable for the presence of microbial species [52]. Previous studies reported a positive correlation between the particulate matter concentration and the levels of airborne microorganisms [53]. In the present studies, bacterial counts were correlated with fungal counts on posaconazole. In fact, particles present in the air may be single microorganisms, groups of microorganisms, single or grouped spores, or fragments of organisms [54]. Overall, bioburden indoors can originate from outdoor air or from humans, e.g., building occupants or visitors, and can vary greatly depending on their activities [55]. Kitchens and living rooms revealed higher fungal counts when compared with bedrooms. This is likely because vegetables and fruits, which are generally prepared in the kitchen, can have an important role as fungal contamination sources [56]. The living room is where most of the visitors and dwelling occupants spend most of the time and this can impact the fungal counts, since human activities have influence on fungal profiles [57]. The fact that occupants spend more time at home in the winter season can also justify the increased fungal contamination in the kitchen [58]. However, the trend of bacterial contamination was opposite to that of fungi, presenting higher counts in the summer season. This can happen due to substrate competition between fungi and bacteria that can boost bacteria and restrict fungi dissemination [59].

Although correlated in the counts, MEA and DG18 presented a different distribution with respect to the species of observed. These results are in line with previous studies in which both culture media were used to obtain a wider characterization of the fungal diversity [30,39,60]. In fact, MEA is the culture media most applied to samples aiming at assessing indoor contamination. It is mainly suitable for yeasts and filamentous fungi, since it contains a high concentration of maltose and other saccharides as energy sources [61]. DG18 is more recently indicated as a better alternative for colony counting and to obtain higher diversity of genera, since this medium also contains dichloran, which inhibits spreading of fungi belonging to Mucorales order [22,62] and restricts the colony size of other genera [62]. Both culture media features justify the differences between the most prevalent fungi in the same sample.

*Aspergillus* sections were detected by molecular tools in nine samples from the winter season. In eight of them, it was not possible to identify the section detected with culture-based methods. This finding corroborates the need to apply both methods in parallel to achieve a better characterization of *Aspergillus* sections, thus overcoming the limitations of each method [2,22,29,30,31,63,64]. Indeed, culture-based methods provide information on the viable/culturable form required to estimate health risks, as it affects biological mechanisms, such as the cytotoxic and inflammatory responses [65], while molecular tools allow a rapid identification and are being gradually used to obtain data on the microbial biodiversity in different indoor environments [63].

As mentioned above, lower bacterial loads in winter, associated with higher amounts of time spent indoors during the cold season, may favor the proliferation of fungi in dwellings, as it was observed in this study. The most frequent fungal species in VOR in samples from the winter campaign were *Penicillium* sp., followed by *Cladosporium* sp., *Chrysosporium* sp., and *Aspergillus* sp., of which the most abundant were sections *Circumdati* and *Nidulantes.* These fungi agree relatively well with those observed in MEA and DG18.

The correlation between the presence of fungi in regular media (MEA and DG18) and azole-supplemented media (ITR, VOR, POS) might indicate some reduced susceptibility to antifungal drugs among the collected species in domestic environments. Several studies describe azole resistance as an emerging problem worldwide, including in Europe, challenging the treatment of azole-resistant Aspergillus disease, mainly caused by *Aspergillus* section *Fumigati* [33,34,66,67,68]. No active surveillance for azole resistance is performed in indoor air quality studies in Portugal and guidelines on how to perform it in complex and composite samples (such as environmental samples) are lacking. We have adapted the EUCAST referential (not well validated for azole resistance detection in fungal species other than *Aspergillus* section *Fumigati*) and have used a four-plate agar system to screen the resistance phenotype of fungal species collected in the environment, mostly by passive sampling, as in the present study. Although this approach does not allow to establish a single resistance phenotype per fungal species, it is a broad and feasible strategy for resistance surveillance campaigns, which even enables the detection of unknown resistance mechanisms that might escape molecular detection [31,69].

On the other hand, the fact that higher values on POS were related to higher values of Cq suggests that fungal species with some reduced susceptibility to posaconazole might be from *Aspergillus* sp., although not observed by culture-based methods. This aspect reinforces, once more, the importance of an algorithm of combined methods (molecular and culture-based) for an accurate fungal assessment in the environment, as the one suggested previously to be applied in occupational environments with azole pressure [31].

## 5. Conclusions

The use of passive sampling methods to assess settleable dust and bioburden allowed having a wider pool of dwellings and sampling locations. Dust loading rates and bioburden presented a wider range in the summer season. However, fungal contamination increased in winter, while bacterial contamination decreased. *Aspergillus* sections *Circumdati* and *Nidulantes* were observed in VOR, as well as in MEA and DG18.

Overall, this study reinforces the importance of applying:

(a) Passive sampling methods in campaigns to assess sedimentable dust and bioburden in dwellings;
(b) MEA and DG18 when using culture-based methods to assess fungi;
(c) In parallel, with culture methods, molecular tools targeting the most suitable indicators of fungal contamination indoors;
(d) Azole resistance screening to unveil azole resistance detection in fungal species besides *Aspergillus* section *Fumigati*.

## Figures and Tables

**Table 1:** Characteristics of the dwellings where dust sampling took place.

Dwelling	Type	Location	Number of Occupants	Wood Heating	Plants	Floor Bedroom	Rugs Bedroom	Floor Living Room	Rugs Living Room	Floor Kitchen	Rugs Kitchen	Exhaust Kitchen	Smokers	Pets
1	Apartment	Urban	5	No	No	Wood	Yes	Marble	Yes	Tile	No	Yes	No	No
2	Apartment	Urban	4	Yes	Yes	Wood	Yes	Wood	Yes	Tile	No	Yes	No	No
3	Apartment	Urban	4	Yes	No	Tile	Yes	Tile	No	Tile	No	No	No	No
4	Apartment	Urban	3	No	No	Wood	Yes	Tile	No	Tile	No	Yes	No	No
5	Apartment	Urban	1	No	No	Parquet	Yes	Parquet	Yes	Vinyl	No	Yes	No	No
6	Apartment	Urban	2	No	Yes	Parquet	Yes	Parquet	Yes	Tile	Yes	Yes	Yes (Living room)	No
7	Detached house	Rural	3	Yes	Yes	Wood	Yes	Tile	Yes	Tile	Yes	Yes	No	No
8	Apartment	Urban	4	Yes	Yes	Wood	No	Wood	Yes	Granite	No	Yes	No	No
9	Apartment	Urban	4	No	No	Wood	No	Wood	Yes	Granite	Yes	Yes	No	Yes (1 Bird)
10	Detached house	Rural	3	No	Yes	Wood	Yes	Tile	Yes	Tile	Yes	Yes	No	Yes (1 Cat)
11	Apartment	Suburban	2	No	Yes	Wood	Yes	Carpet	Yes	Tile	Yes	Yes	No	Yes (1 Hamster)
12	Detached house	Rural	3	Yes	Yes	Wood	Yes	Wood	Yes	Granite	Yes	Yes	No	No
13	Apartment	Urban	4	No	Yes	Wood	Yes	Tile	Yes	Tile	Yes	Yes	Yes (Kitchen)	Yes (1 Dog)
14	Detached house	Rural	4	No	Yes	Wood	Yes	Tile	Yes	Tile	Yes	No	No	Yes (4 Cats)
15	Apartment	Urban	3	Yes	Yes	Wood	Yes	Wood	Yes	Tile	Yes	Yes	No	Yes (1 Dog)
16	Terraced house	Urban	5	No	No	Wood	Yes	Wood	Yes	Tile and Wood	Yes	Yes	No	No
17	Detached house	Suburban	4	Yes	Yes	Wood	No	Tile	Yes	Tile	No	Yes	No	No
18	Terraced house	Urban	2	Yes	Yes	Wood	Yes	Tile	Yes	Tile	No	Yes	No	No
19	Detached house	Suburban	3	No	Yes	Wood	Yes	Tile	Yes	Tile	No	Yes	No	No
20	Apartment	Suburban	2	Yes	Yes	Parquet	Yes	Parquet	Yes	Tile	Yes	Yes	Yes (Kitchen)	No
21	Semi-detached house	Rural	4	No	Yes	Wood	Yes	Wood	Yes	Tile	Yes	Yes	No	No
22	Apartment	Urban	3	Yes	Yes	Wood	Yes	Wood	Yes	Tile	No	Yes	No	No
23	Apartment	Urban	2	Yes	Yes	Wood	Yes	Wood	Yes	PVC	No	Yes	No	No

**Table 2:** Sequence of primers and TaqMan probes used for Real Time PCR.

*Aspergillus* Sections Targeted	Sequences	Reference
*Flavi* (Toxigenic Strains)		
Forward Primer	5′-GTCCAAGCAACAGGCCAAGT-3′	[39]
Reverse Primer	5′-TCGTGCATGTTGGTGATGGT-3′	
Probe	5′-TGTCTTGATCGGCGCCCG-3′	
*Fumigati*		
Forward Primer	5′-CGCGTCCGGTCCTCG-3′	[40]
Reverse Primer	5′-TTAGAAAAATAAAGTTGGGTGTCGG-3′	
Probe	5′-TGTCACCTGCTCTGTAGGCCCG-3′	
*Circumdati*		
Forward Primer	5′-CGGGTCTAATGCAGCTCCAA-3′	[41]
Reverse Primer	5′-CGGGCACCAATCCTTTCA-3′	
Probe	5′-CGTCAATAAGCGCTTTT-3′	
*Nidulantes*		
Forward Primer	5′-CGGCGGGGAGCCCT-3′	[42]
Reverse Primer	5′-CCATTGTTGAAAGTTTTGACTGATcTTA-3′	

**Table 3:** Seasonal dust loadings (mean standard deviation) in dwelling of the district of Aveiro. Values are given in mg/m^2^/day.

	Winter	Summer
Global mean	4.29 ± 4.51	5.70 ± 2.70
Bedrooms	4.03 ± 4.04	6.26 ± 2.74
Living rooms	3.55 ± 4.28	5.08 ± 2.63
Kitchens	5.38 ± 5.11	6.19 ± 2.68

**Table 4:** Comparison between dust loads (µg/cm^2^/day) in summer and winter.

				Ranks		Test Statistics ^d^	
			N	Mean Rank	Sum of Ranks	Z	*p*
Dust loadings Winter–Dust loadings Summer		Negative Ranks	16 ^a^	35.22	563.50	−3.187 ^d^	0.001 *
		Positive Ranks	48 ^b^	31.59	1516.50		
		Ties	0 ^c^				
		Total	64				
Bedroom	Dust loadings Winter–Dust loadings Summer	Negative Ranks	4 ^a^	10.63	42.50	−2.538 ^e^	0.011 *
		Positive Ranks	17 ^b^	11.09	188.50		
		Ties	0 ^c^				
		Total					
Living room	Dust loadings Winter–Dust loadings Summer	Negative Ranks	5 ^a^	10.00	50.00	−2.053 ^d^	0.040 *
		Positive Ranks	15 ^b^	10.67	160.00		
		Ties	0 ^c^				
		Total					
Kitchen	Dust loadings Winter–Dust loadings Summer	Negative Ranks	6 ^a^	14.50	87.00	−1.282 ^e^	0.200
		Positive Ranks	16 ^b^	10.38	166.00		
		Ties	0 ^c^				
		Total					

^a^ Dust loadings Winter < Dust loadings Summer. ^b^ Dust loadings Winter > Dust loadings Summer. ^c^ Dust loadings Winter = Dust loadings Summer. ^d^ Based on negative ranks. ^e^ Based on positive ranks. * Statistically significant differences at the 5% significance level.

**Table 5:** Distribution of bacterial contamination on Electrostatic Dust Collector (EDC).

**Summer**	
	**Mean (SD) CFU/m^2^/Day**
TSA	6.03 × 10^3^ (1.84 × 10^4^)
VRBA	1.33 × 10^2^ (5.50 × 10^2^)
**Winter**	
	**Mean (SD) CFU/m^2^/day**
TSA	5.17 × 10^1^ (1.73 × 10^2^)
VRBA	1.15 × 10^1^ (9.81 × 10^1^)

**Table 6:** Fungal contamination found in each season.

**Summer**					
**MEA**			**DG18**		
**Fungi**	**CFU/m^2^/Day**	**%**	**Fungi**	**CFU/m^2^/Day**	**%**
*Penicillium* sp.	1.45 × 10^3^	58.1	*Cladosporium* sp.	1.45 × 10^3^	46.3
*C. sitophila*	2.48 × 10^2^	9.92	*Penicillium* sp.	1.09 × 10^3^	34.9
*Cladosporium* sp.	1.92 × 10^2^	7.65	*C. sitophila*	2.25 × 10^2^	7.16
*Aspergillus* sp.	1.67 × 10^2^	6.67	*Aspergillus* sp.	1.99 × 10^2^	6.35
Other species	4.43 × 10^2^	17.7	Other species	1.68 × 10^2^	5.37
**TOTAL**	2.50 × 10^3^	100	TOTAL	3.14 × 10^3^	100
**Winter**					
**MEA**			**DG18**		
**Fungi**	**CFU/m^2^/day**	**%**	**Fungi**	**CFU/m^2^/day**	**%**
*Penicillium* sp.	1.47 × 10^3^	59.1	*Penicillium* sp.	1.69 × 10^3^	52.2
*Aspergillus* sp.	3.22 × 10^2^	13.0	*Cladosporium* sp.	7.11 × 10^2^	21.9
*Fusarium* sp.	2.21 × 10^2^	8.90	*Chrysosporium* sp.	5.49 × 10^2^	16.9
*Cladosporium* sp.	2.17 × 10^2^	8.75	*Aspergillus* sp.	1.61 × 10^2^	4.98
Other species	2.55 × 10^2^	10.3	Other species	1.30 × 10^2^	4.00
**TOTAL**	2.48 × 10^3^	100	TOTAL	324 × 10^4^	100

**Table 7:** Fungal burden found in each home location, per season.

Season		ITR		VOR		POS	
	Location	CFU/m^2^/Day	%	CFU/m^2^/Day	%	CFU/m^2^/Day	%
summer	Bedroom	2.58 × 10^3^	14.1	7.82 × 10^3^	21.5	1.43 × 10^3^	28.2
	Kitchen	9.04 × 10^3^	49.2	1.66 × 10^4^	45.7	1.88 × 10^3^	37.0
	Living room	6.74 × 10^3^	36.7	1.19 × 10^4^	32.8	1.77 × 10^3^	34.8
	TOTAL	1.84 × 10^4^	100	3.64 × 10^4^	100	5.09 × 10^3^	100
winter	Bedroom	1.77 × 10^2^	0.9	1.28 × 10^4^	15.8	4.56 × 10^3^	21.2
	Kitchen	5.08 × 10^3^	25.1	4.54 × 10^4^	56.1	1.41 × 10^4^	65.5
	Living room	1.50 × 10^4^	74.1	2.28 × 10^4^	28.1	2.85 × 10^3^	13.3
	TOTAL	2.03 × 10^4^	100	8.11 × 10^4^	100	2.15 × 10^4^	100

**Table 8:** Fungal levels found in EDCs during azole screening, per season.

		SDA		ITR		VOR		POS	
Season	Fungi	CFU/m^2^/Day	%	CFU/m^2^/Day	%	CFU/m^2^/Day	%	CFU/m^2^/Day	%
summer	*Aspergillus* sp.	1.26 × 10^3^	13.9	n.d.	0.0	n.d.	0.0	n.d.	0.0
	*Chrysosporium* sp.	4.71 × 10^1^	0.5	3.73 × 10^1^	3.0	4.62 × 10^1^	1.0	0.83 × 10^1^	7.6
	*Cladosporium* sp.	1.04 × 10^2^	1.1	2.50 × 10^1^	2.0	5.45 × 10^1^	1.2	n.d.	0.0
	*Fusarium* sp.	0.83 × 10^1^	0.1	0.83 × 10^1^	0.7	0.41 × 10^1^	0.1	0.41 × 10^1^	3.8
	*Mucor* sp.	2.07 × 10^3^	22.9	n.d.	0.0	2.03 × 10^3^	45.1	n.d.	0.0
	*Penicillium* sp.	4.46 × 10^2^	4.9	1.99 × 10^2^	15.9	3.05 × 10^2^	6.8	9.64 × 10^1^	88.6
	*Rhizopus* sp.	4.07 × 10^3^	45.0	9.84 × 10^2^	78.5	2.05 × 10^3^	45.6	n.d.	0.0
	Other species	1.04 × 10^3^	11.5	n.d.	0.0	0.43 × 10^1^	0.1	n.d.	0.0
	TOTAL	9.05 × 10^3^	100	1.25 × 10^3^	100	4.50 × 10^3^	100	1.09 × 10^2^	100
Winter	*Aspergillus* sp.	1.75 × 10^2^	4.6	n.d.	0.0	0.72 × 10^1^	1.3	n.d.	0.0
	*Chrysosporium* sp.	1.12 × 10^2^	3.0	0.17 × 10^1^	3.2	2.02 × 10^1^	3.8	n.d.	0.0
	*Cladosporium* sp.	2.44 × 10^1^	0.6	0.90 × 10^1^	17.5	2.25 × 10^2^	42.0	1.23 × 10^2^	70.2
	*Fusarium* sp.	0.35 × 10^1^	0.1	n.d.	0.0	n.d.	0.0	0.18 × 10^1^	1.0
	*Mucor* sp.	9.28 × 10^2^	24.3	n.d.	0.0	n.d.	0.0	n.d.	0.0
	*Penicillium* sp.	8.36 × 10^2^	21.9	4.06 × 10^1^	79.3	2.84 × 10^2^	52.9	5.02 × 10^1^	28.8
	*Rhizopus* sp.	1.71 × 10^3^	44.8	n.d.	0.0	n.d.	0.0	n.d.	0.0
	Other species	2.56 × 10^1^	0.7	n.d.	0.0	n.d.	0.0	n.d.	0.0
	TOTAL	3.81 × 10^3^	100	5.13 × 10^1^	100	5.37 × 10^2^	100	1.75 × 10^2^	100

n.d., not detected.

**Table 9:** Molecular detection of *Aspergillus* sections *Fumigati* and *Nidulantes*.

*Aspergillus* Section Detected	Sample Origin	CFU.m^−2^ (in MEA/DG18)	C_q_
*Fumigati*	Bedroom	0/0	32.6
		0/0	34.9
	Living room	1.06 × 10^2^/0	33.8
		0/0	31.1
	Kitchen	0/0	30.3
		0/0	31.4
		0/0	29.7
*Nidulantes*	Bedroom	0/0	38.1
	Kitchen	0/0	37.7

**Table 10:** Relationships between bacterial counts, fungal counts (MEA and DG18), azole resistance (itraconazole (ITR), voriconazole (VOR), and posaconazole (POS) media), and molecular tools (Cq) established by Spearman correlations.

			Summer								
			Days	Bacteria (CFU/m^−2^/Day)		Fungi (CFU/m^−2^/Day)		Fungi in Azole-Screening Media (CFU/m^−2^/day)			Molecular Tools
				TSA	VRBA	MEA	DG18	ITR	VOR	POS	Cq
summer	Dust loadings (µg/cm^2^/day		0.163	0.151	−0.026	0.159	0.022	0.190	0.049	0.013	
	Days			0.054	0.103	−0.061	0.162	0.052	−0.034	0.014	
	Bacteria (CFU/m^2^/day)	TSA			0.233	0.096	0.122	−0.016	0.051	0.168	
		VRBA				0.129	0.062	−0.071	−0.025	−0.072	
	Fungi (CFU/m^2^/day)	MEA					0.430 **	0.064	0.103	−0.146	
		DG18						0.077	0.041	−0.121	
	Fungi in azole-screening media (CFU/m^2^/day)	ITR							0.213	0.150	
		VOR								0.053	
		POS									
**Winter**											
winter	Dust loadings (µg/cm^2^/day		0.174	0.397 **	0.161	0.087	0.171	0.124	0.119	0.244 *	0.650
	Days			0.060	−0.083	0.013	0.087	0.028	−0.060	−0.073	−0.522
	Bacteria (CFU/m^2^/day)	TSA			0.305 **	0.074	0.144	−0.128	0.184	−0.008	0.609
		VRBA				−0.136	−0.181	−0.129	0.059	0.144	
	Fungi (CFU/m^2^/day)	MEA					0.710 **	0.380 **	0.382 **	0.281 *	−0.092
		DG18						0.246 *	0.419 **	0.213	−0.360
	Fungi in azole-screening media (CFU/m^2^/day)	ITR							0.180	0.312 **	0.525
		VOR								0.463 **	0.424
		POS									0.772 *

** Correlation is significant at the 0.01 level (2-tailed). * Correlation is significant at the 0.05 level (2-tailed).

**Table 11:** Comparison between summer and winter for dust loads, bacterial and fungal counts, and azole screening resistance.

			Ranks		Test Statistics ^v^	
		N	Mean Rank	Sum of Ranks	z	*p*
TSA Winter (CFU/m^2^/day)-TSA Summer (CFU/m^2^/day)	Negative Ranks	58 ^a^	29.50	1711.00	−6.624 ^w^	0.000 *
	Positive Ranks	0 ^b^	0.00	0.00		
	Ties	6 ^c^				
	Total	64				
VRBA Winter (CFU/m^2^/day)-VRBA Summer (CFU/m^2^/day)	Negative Ranks	10 ^d^	8.50	85.00	−2.761 ^w^	0.006 *
	Positive Ranks	3 ^e^	2.00	6.00		
	Ties	53 ^f^				
	Total	66				
MEA Winter (CFU/m^2^/day)-MEA Summer (CFU/m^2^/day)	Negative Ranks	16 ^g^	13.63	218.00	−5.584 ^x^	0.000 *
	Positive Ranks	49 ^h^	39.33	1927.00		
	Ties	0 ^i^				
	Total	65				
DG18 Winter (CFU/m^2^/day)-DG18 Summer (CFU/m^2^/day)	Negative Ranks	8 ^j^	15.13	121.00	−6.073 ^x^	0.000 *
	Positive Ranks	55 ^k^	34.45	1895.00		
	Ties	3 ^l^				
	Total	66				
ITR Winter (CFU/m^2^/day)-ITR Summer (CFU/m^2^/day)	Negative Ranks	15 ^m^	14.97	224.50	−1.245 ^w^	0.213
	Positive Ranks	11 ^n^	11.50	126.50		
	Ties	40 ^o^				
	Total	66				
VOR Winter (CFU/m^2^/day)-VOR Summer (CFU/m^2^/day)	Negative Ranks	23 ^p^	25.39	584.00	−1.558 ^x^	0.119
	Positive Ranks	32 ^q^	29.88	956.00		
	Ties	11 ^r^				
	Total	66				
POS Winter (CFU/m^2^/day)-POS Summer (CFU/m^2^/day)	Negative Ranks	10 ^s^	10.70	107.00	−1.229 ^x^	0.219
	Positive Ranks	14 ^t^	13.79	193.00		
	Ties	42 ^u^				
	Total	66				

^a^ TSA Winter < TSA Summer. ^b^ TSA Winter > TSA Summer. ^c^ TSA Winter = TSA Summer. ^d^ VRBA Winter < VRBA Summer. ^e^ RB Winter > RB Summer. ^f^ RB Winter = RB Summer. ^g^ MEA Winter < MEA Summer. ^h^ MEA Winter > MEA Summer. ^i^ MEA Winter = MEA Summer. ^j^ DG18 Winter < DG18 Summer. ^k^ DG18 Winter > DG18 Summer. ^l^ DG18 Winter = DG18 Summer. ^m^ ITRA Winter < ITRA Summer. ^n^ ITRA Winter > ITRA Summer. ^o^ ITR Winter = ITRA Summer. ^p^ VOR Winter < VOR Summer. ^q^ VOR Winter > VOR Summer. ^r^ VOR Winter = VOR Summer. ^s^ POS Winter < POS Summer. ^t^ POS Winter > POS Summer. ^u^ POS Winter = POS Summer. ^v^ Wilcoxon Signed Ranks Test. ^w^ Based on positive ranks. ^x^ Based on negative ranks. * statistically significant differences at the 5% significance level.
